# Delayed gratification in the adult brain

**DOI:** 10.7554/eLife.59786

**Published:** 2020-07-21

**Authors:** Gerd Kempermann

**Affiliations:** 1 German Center for Neurodegenerative Diseases Dresden, TU DresdenDresdenGermany; 2 Center for Regenerative Therapies Dresden, TU DresdenDresdenGermany

**Keywords:** immature neurons, neocortex, doublecortin, brain size, mammals, Other

## Abstract

Some immature neurons in the cerebral cortex of mammals might wait for years before they become activated and finish their development.

**Related research article** La Rosa C, Cavallo F, Pecora A, Chincarini M, Ala U, Faulkes CG, Nacher J, Cozzi B, Sherwood C, Amrein I, Bonfanti L. 2020. Phylogenetic variation in cortical layer II immature neuron reservoir of mammals. *eLife* **9**:e55456. doi: 10.7554/eLife.55456

The history of adult neurogenesis – the ability of the developed brain to generate new neurons – is somewhat convoluted. In ground-breaking experiments at MIT in the early 1960s, Joseph Altman and Gopal Das injected thymidine that had been labeled with tritium into the brains of young rats: when labeled thymidine was later observed in neurons, the obvious explanation was that these neurons had been generated by the adult brain (Altman and Das, 1965). Altman and Das had speculated that ‘some’ progenitor cells must have been responsible, but it was only with the discovery of neural stem cells in the early 1990s that the origins of adult neurogenesis became clear (Cameron et al., 1993; Gage, 2000).

Many fish and birds can form new neurons from stem cells in their entire brain and throughout their life (Kaslin et al., 2008), but mammals seem to lack this ability – or have only limited potential to do so. The reduced regenerative capacity that might arise from this has been interpreted as the price that mammals pay for the unsurpassed complexity of the neocortex.

Neuronal development in the embryo is mainly a preset process that is defined by decisive cell divisions at the stem cell level. However, extrinsic regulation and behavioral activity have extensive influence in the adult brain, and control adult neurogenesis in mammals at many levels. But what if new neurons could also develop from other cells within the neurogenic lineage, such as dormant intermediate cells waiting to become activated? A similar mechanism can, for example, be found in human oocytes, where germ cell proliferation ceases well before sexual maturity and further development originates from dormant cells many years later.

Now, in eLife, Luca Bonfanti and colleagues – including Chiara La Rosa as first author – report new insights into this theory by describing immature neurons in the cortex of different mammals (La Rosa et al., 2020). Although first classified in 2008, we still know little about these cortical immature neurons, especially about their functional relevance (Gómez-Climent et al., 2008; Bonfanti and Nacher, 2012). These cells are created before birth but seem to be arrested in a state that lacks the final leap in maturation required for full functionality. It is thought that they could serve as a dormant reservoir of cells beyond the proliferative precursor cell stage, and as a delayed starting point for neurogenesis dissociated from stem cell division. Neurogenesis without cell division would presumably be faster and safer than the risky process of proliferation followed by differentiation (which requires many checkpoints in its early stages).

The researchers – who are based at the University of Turin and other research institutes in Italy, Spain, Switzerland, the UK and the US – studied the distribution and properties of cortical immature neurons in 80 brains across a phylogenetically diverse range of mammal species. They discovered that the larger the brain, the greater the number of immature neurons in the second cortical layer just below the brain surface (and thus far away from the niche for precursor cells near the ventricular wall). Moreover, brains with more folds contained greater densities of immature neurons across their neocortex (Figure 1).

**Figure 1.**
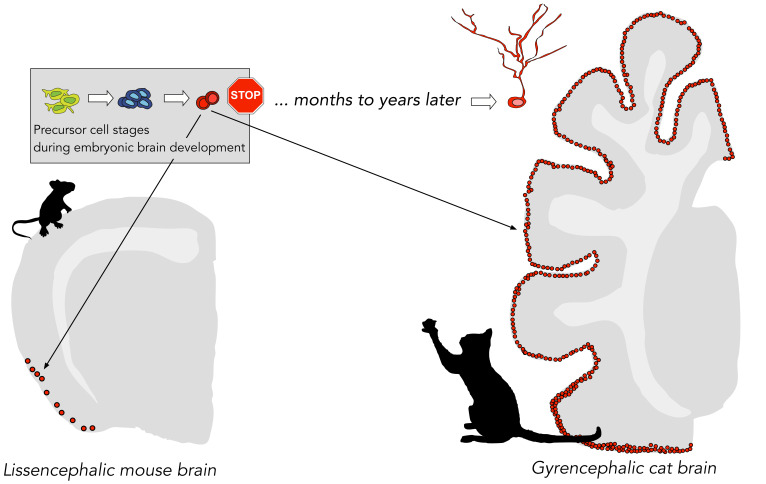
Distribution of immature cortical neurons in different mammals. A particular type of adult neurogenesis in mammals is thought to take place in the cortex through a reservoir of immature cortical neurons, which are created before birth (green, blue and red cells) and remain dormant (indicated by the stop sign) until their maturation into functional neurons might be activated many years later. La Rosa et al. discovered that mammals with larger brains and denser brain folds (e.g., a cat) have more immature cortical neurons than mammals with smaller brains (e.g., a mouse).

La Rosa et al. suggest that these cells provide a reservoir of undifferentiated neurons, which may represent a mechanism for plasticity that varies among mammalian species. Immature cortical neurons could be adaptable and help maintain cognitive processes throughout life. Indeed, previous studies in adult mice revealed that immature neurons in the piriform cortex showed signs of both immaturity and synaptic plasticity (Klempin et al., 2011). Their further development remains to be proven, but is a fascinating hypothesis and has interesting implications for the role of brain plasticity in health and disease.

Dormant immature neurons could also play a role in adult neurogenesis of the human hippocampus (Kempermann et al., 2018), which shares many similarities in learning and memory with its rodent counterpart, but might show different dynamics in adult neurogenesis. Consequently, adult mammals with large brains – especially humans – might not necessarily lack adult neurogenesis (or have very low levels of it); they may simply delay the maturation of new neurons for decades. In theory, this process could allow quicker adaptive responses than adult neurogenesis, which has to be stimulated at the stem cell level.

The study of La Rosa et al. supports such speculations, inviting further research on the exact nature of these immature neurons: How do they maintain their supposedly dormant state? How do they end this state, if ever? And what is their ultimate fate and function? Maybe they are not as dormant as we are tempted to think?
